# Measuring the processes of interdisciplinary team collaboration: Creating valid measures using a many-facet Rasch model approach

**DOI:** 10.1017/cts.2022.472

**Published:** 2022-10-06

**Authors:** Jue Wang, Soyeon Ahn, Susan E. Morgan

**Affiliations:** 1 Department of Psychology, University of Science and Technology of China, Hefei, China; 2 Department of Educational and Psychological Studies, University of Miami, Miami, FL, USA; 3 Department of Communication Studies, University of Miami, Miami, FL, USA

**Keywords:** Science of team science, team science assessment, Rasch measurement theory, reliability, validity, fairness

## Abstract

**Introduction:**

The science of team science (SciTS) is an emerging research area that studies the processes and outcomes of team-based research. A well-established conceptual framework and appropriate methodology for examining the effectiveness of team science are critically important for promoting and advancing collaborative and interdisciplinary research. Although many instruments have been developed and used in the SciTS field, psychometric evidence has not been routinely assessed or reported for these scales. In addition, commonly used psychometric methods were mainly limited to internal consistency and factor analysis. To fill the gaps, this study introduces a framework based on Rasch measurement theory for creating and evaluating measures for team sciences.

**Methods:**

We illustrate the application of Rasch measurement theory through the creation of valid measures to evaluate the processes of interdisciplinary scientific teams. Data were collected from 16 interdisciplinary teams through a university-wide initiative for promoting interdisciplinary team collaboration. Psychometric evidence based on a many-facet Rasch model was obtained for assessing the quality of the measures.

**Results:**

The interdisciplinary teams differed in their clarity measures. Significant differences were also found between gender groups, racial groups, and academic ranks. We reported the reliability of measures and identified items that do not fit the model and may present potential threat to validity and fairness of SciTS measures.

**Conclusion:**

This study shows the great potential of using Rasch measurement theory for developing and evaluating SciTS measures. Applying Rasch measurement theory produces objective measures that are comparable across individuals, interdisciplinary teams, institutions, time, and various demographic groups.

## Introduction

Research conducted by teams has gained its popularity across almost all fields, and it is more frequently cited than single-authored research products [1]. This trend is, on one hand, due to more complex scientific challenges that require research collaboration across disciplines, institutions, time, and geographic locations; on the other hand, rapid developments in science, technology, and communication techniques allow researchers to collaborate virtually [2].

Although collaboration in teams can accelerate the advancement of scientific research and produce greater impact [1,3,4], team collaboration is associated with significant challenges. These include extra effort required for communication across disciplines, ambiguity in research goals, processes, and negotiating individual roles in teams. Understanding the complex issues faced by scientific teams has necessitated the development of a cross-disciplinary field – the science of team science (SciTS). This field focuses on identifying the relationship of factors that influence the effectiveness of teams. Empirical work in SciTS has led to evidence-based approaches that have advanced the research of team science and which have led to improved approaches to interdisciplinary team development [2,5,6]. However, the continued development of SciTS requires improved measurement methods to accurately assess the factors that influence the effectiveness of scientific teams. In other words, the instruments measuring various team characteristics must be accurate and consistent in order to produce valid, reliable, and fair scores.

Tigges *et al.* [7] conducted a systematic review summarizing existing instruments for measuring the quality of team collaboration (e.g., process measures, team trust, and satisfaction). This is, so far, the only study focused on the quality of SciTS measures and provided scientific evidence to call for attention to the item development process and psychometric examination of SciTS measures in published studies. Tigges *et al.* pointed out that SciTS researchers often select items and create the instrument based on the existing surveys. However, items that function well in one setting or in one population may not produce the same degree of reliability and validity in a different context. The users of an instrument should examine the quality of the instrument and how well the items measure the intended construct. This psychometric evidence should be reported in manuscripts that investigate the relationships between variables or that otherwise draw inferences to improve the understanding of the construct.

Moreover, it is critically essential to create accurate measures of the characteristics and attitudes of research teams and compare these teams objectively. However, standard scales are not yet available in SciTS field; large-scale testing across settings and populations is essential to support psychometric arguments [7]. Tigges *et al.*’s [7] examination of the published measures for the quality of collaboration indicated a dominant use of coefficient alpha as the reliability evidence for examining the internal consistency. Among all the reviewed studies, only one (i.e., Mallinson *et al.* [8]) explored modern measurement theory and applied a dichotomous Rasch measurement model for evaluating construct validity, internal consistency, and precision of measures. In addition, score fairness as an important aspect of psychometric quality has not been reported or discussed for SciTS measures in existing literature. The concept of score fairness and how it applies to team science measures will be explicated below.

Rasch measurement models [9] use modern measurement techniques to establish standard scales that allow researchers to compare scores on measures objectively. One advantage of utilizing Rasch modeling is the ability to obtain scaled scores. Scaled scores share equal intervals, while raw scores are at best ordinal. With the use of raw scores (either the sum score or the average), we can only make inferences about the rank order of the research teams, for example, Team A has a higher score on trust than Team B, and Team B is higher on trust than Team C. Statistically, no addition or subtraction can be performed on raw team scores in a way that yields valid results, while scaled scores can be treated as continuous measures and can also be used to conduct various general linear modeling analyses (e.g., Analysis of Variance, Regression, and multilevel modeling).

### Purpose of the Study

In this study, we aim to demonstrate how a modern measurement framework can be utilized for examining team science measures using empirical data. A many-facet Rasch model, based on Rasch measurement theory, is used to calibrate an underlying scale and obtain latent measures. Validity, reliability, and fairness-in-measurement issues are first discussed using this framework. Then, we use a real-world case with data collected through one university’s internal pilot funding mechanism for interdisciplinary research to show how to make comparisons between research teams and different demographic groups using latent measures as well as ways to evaluate psychometric properties of team science measures. In addition to creating valid measures that can be used immediately by SciTS researchers, it is our intention to help advance an understanding of the measurement procedure that can be used to create other valid measures to help researchers clarify the best predictors, processes, and outcomes of collaborative research teams. This approach to measurement can also support administrators and policymakers make well-informed decisions about research teams that should receive investments of internal funding based on valid predictions of which teams are the most likely to succeed.

## Theoretical Framework

**Fig. 1.**
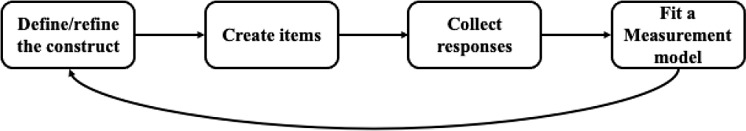
Process of scale development.

Based on Lazarsfeld’s [10] description of the measurement process, researchers have developed a theoretical framework for constructing invariant measures using four key processes – (a) defining the latent variable, (b) creating/selecting items according to the definition of a construct, (c) collecting item responses (e.g., rating responses and test scores), and (d) making inferences using an appropriate psychometric model (presented in Fig. 1; [11–13]). Inferences based on measures of a latent variable can further improve the understanding of the construct, which connects these four components to create an iterative process. To evaluate the measurement procedure and support score inferences, we need to obtain and report validity, reliability, and fairness evidence [14].

**Fig. 2.**
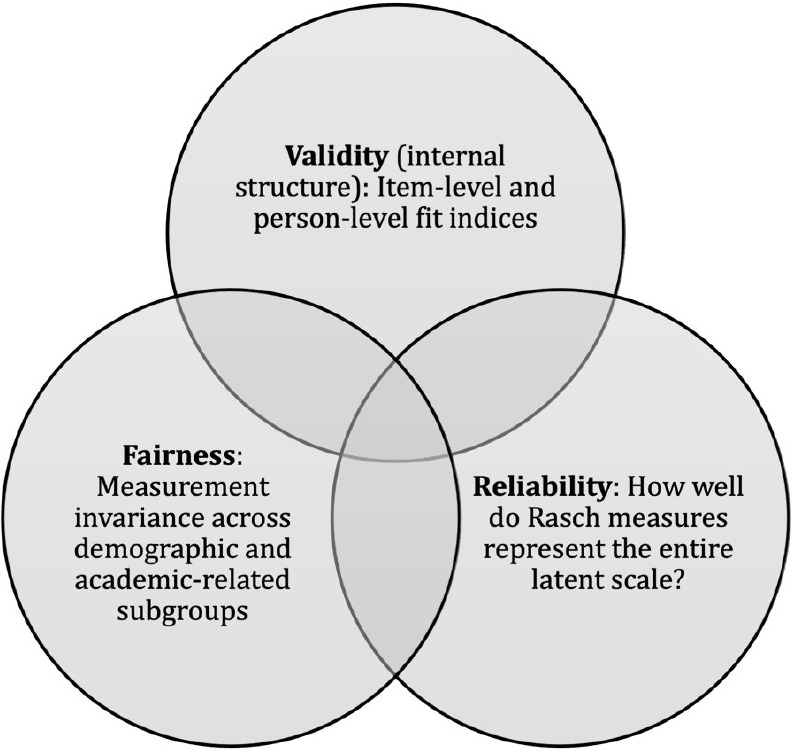
Three foundational areas for evaluating psychometric quality of team science measures.

The choice of a specific measurement model is critical in producing valid and reliable measures. Various disciplines such as science education, music education, literacy education, public health, food insecurity, and other social science areas have applied Rasch measurement theory. Because the SciTS field focuses on collaborative, team-based research processes, we study psychological factors including behaviors, cognition, and emotions of collaborators that may influence team effectiveness. Rasch measurement theory can be used to establish validity, reliability, and fairness evidence (Fig. 2).

### Validity

Validity refers to the degree to which evidence and theory support the interpretation of scores or measures for proposed uses of measurement instruments [14]. We can create an integrated validity argument using evidence based on (a) item content, (b) response process, (c) internal structure, (d) relations to other variables, and (e) outcomes of assessment, termed consequential validity.

The evidence based on the content can be established by defining the underlying construct and specifying the item development procedures. The degree to which the items represent the measuring construct as well as the extent to which the cognitive processes of respondents are consistent with the interpretation of scores provide relevant validity evidence of internal structure. The relationship of a construct to relevant external variables that are measured by other instruments can establish predictive and criterion validity. Consequential validity refers to the intended and unintended consequences of making inferences using measures. Qualitative methods, including survey, interview, think-aloud, and focus group discussion, can be used to obtain evidence for the validity of item content and response process.

Rasch measurement models provide item-level and person-level fit indices, such as the Infit and Outfit mean square statistics, for evaluating the fit between model and data. The fit indices provide diagnostic information for identifying misfit individual on each facet (e.g., person, item, or research team) and support the validity arguments of internal structure.

### Reliability

Reliability or precision, broadly defined, refers to the consistency of scores across replications of an evaluation procedure. While the coefficient alpha, which is based on the Classical Test Theory, has been widely used for evaluating internal consistency among items, its calculation requires that scores be interval measures. Thus, survey responses or raw scores can at most be viewed as ordinal ratings which means that the use of coefficient alpha mistreats ordinal ratings as interval measures [15,16]. Thus, most measures used in SciTS studies are being used in ways that are less than ideal.

Based on Rasch measurement theory, a *reliability of separation* index can be calculated using latent measures; such latent measures share equal intervals along the underlying continuum [17]. We can obtain a *reliability of separation* index for each facet, reflecting how distinct the latent measures are along the scale. The values range from 0 to 1. Higher reliabilities are preferred because they indicate a good presentation of Rasch scores across the entire range of the latent scale.

### Fairness

Fairness of scores is a fundamental issue in measurement. Fairness refers to consistency in the interpretation of responses to survey items across individuals and contexts. For interdisciplinary teams, team members with different backgrounds and experiences may perceive and interpret items differently. Stakes can be high on this issue, particularly when funding or administrative support decisions are based on the results of these measures. Therefore, ensuring the fairness of the measurement process is critically important. It is also crucial for policymakers and university administrators to make decisions grounded in valid interpretations of empirical data.

Fairness emphasizes measurement invariance across subgroups that differ by individual demographic and academic-related variables (e.g., gender, academic ranks, and race). Other factors can present potential threats to fairness in SciTS, including a lack of clarity in the instructions, unrelated complexity, and unnecessary language demands, which can systematically bias the scores of specific subgroups. If an item elicits different meanings to individuals in different subgroups, it creates bias and hampers measurement invariance across subgroups. A good-quality instrument should provide fair scores to individuals from different subgroups.

To detect any potential bias due to individual items, we can use differential item functioning (DIF) analysis. Based on the Rasch model, a DIF analysis compares group differences through *t*-tests on item Rasch scores. A significant *t*-statistic indicates the existence of DIF. Any item that presents DIF may be a potential threat to the fairness of score decisions and should be re-examined before further uses.

In summary, introducing and advancing modern measurement techniques to develop and evaluate the processes of scientific teams can enhance the validity, reliability, and fairness of measurement.

## Methodology

### Interdisciplinary Research Funding Program

U-LINK (**U**niversity of Miami **L**aboratory for **IN**tegrative **K**nowledge) is an evidence-based interdisciplinary pilot research funding program developed according to recommendations offered by researchers working in the area of “the science of team science” (SciTS). While U-LINK is similar to other university pilot funding programs in its focus on grand challenges to society, there are a number of characteristics of U-LINK that make the program unusual relative to other funding programs: (1) meaningful partnerships with university stakeholder groups, including the Clinical Translational Science Institute (CTSI), the Graduate School, and university Libraries; (2) a phased funding model that offers release time (or overload funding) to members of faculty teams to engage in the teaming process (Phase 1) before providing a second phase of funding to a smaller number of teams to establish the feasibility of their approach to help support external funding applications; (3) required participation in an annual team science training program; (4) required engagement of community stakeholders from the earliest stages of research. Please see Morgan *et al.* [6] and https://doi.org/10.33596/ovprrs-19 for a full description of the U-LINK program and evaluation of training program content.

### Participants

U-LINK awardees from 2020 funding cycles participated in this study. Individual members who did not respond to any of the items or did not report complete demographic information were removed from the analysis, resulting in 53 members from 16 research teams as the final sample. About half of the participants were female (*n* = 27), and 26 identified as male. Participants included tenure-track faculty at all ranks: assistant professors (*n* = 13; 24.5%), associate professors (*n* = 15; 28.3%), and full professors (*n* = 12; 22.6%). There are 13 non-tenure-track faculty (24.5%), and among them, seven are clinical and research professors. Diverse disciplines were represented across STEM and non-STEM fields, including engineering, public health, atmospheric sciences, architecture, communication, English, history, economics, business, psychology, computer science, and interactive media.

### Data Collection Procedures

We collected survey data from U-LINK awardees using Qualtrics. A survey link was emailed to all awardees, followed by at least one follow-up reminder. No incentives for survey completion were provided. This study was determined to be exempt from IRB review because it falls under “process improvement” rather than human subjects research. Data collected in 2020 was used for the current study.

### Variables and Measures

Of the data collected from U-LINK awardees, items measuring the *goal clarity*, *role clarity*, and *process clarity* were used for the analysis. Appendix A1 lists the items included in our questionnaire and the variables of individual characteristics. For the item statements, participants rated their agreement on a 5-point Likert response scale, ranging from 1 = Disagree to 5 = Agree.

#### Goal clarity

Sawyer’s [18] measure of goal and process clarity served as the foundation for this assessment. The items include “*I am clear about my responsibilities on this U-LINK team,*” “*I am confident that I know what the goals are for my U-LINK team*,” and “*I know how my work relates to the overall objectives of my U-LINK team*.”

#### Role clarity

We used two items from Peterson and colleagues’ [19] measurement of role ambiguity, conflict, and overload. These items are “*I know exactly what is expected of me on my U-LINK team*” and “*I know what my responsibilities are on my U-LINK team*.”

#### Process clarity

Three items from Sawyer’s [18] measurement of goal and process clarity were used to assess process clarity. Sample items include “*I know how to go about my work on my U-LINK team*,” “*I know how my team will move forward with its work on our U-LINK project*,” and “*I am confident that my U-LINK team is using the right processes to move forward with its work*.”

#### Individual characteristics

Demographic and personal characteristics included the following: gender (1 – male; 2 – female), ethnicity group (1 – Hispanic or Latino; 2 – Not Hispanic or Latino), racial group (1 – Asian; 2 – Black or African American; 3 – White; 4 – Decline to specify), academic rank title (1 – Assistant Professor; 2 – Associate Professor; 3 – Full Professor; 4 – Clinical Professor/Professor of Practice/Research Professor; 5 – Other) as well as their team number, and whether or not having interdisciplinary experience before (0 – No; 1 – Yes). Please note that the selective categories for racial group are comprehensive on the survey; however, our sample consisted of only three racial groups.

### Model Specification

A Many-Facet Rasch Model (MFRM; [20]) is used to examine the ratings on clarity. The items reflect different aspects of goal clarity, role clarity, and process clarity. The MFRM is expressed as below. We used the FACETS computer program [21] to perform the data analysis. The syntax is included in the Appendix B.
(1)

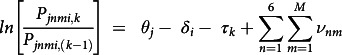




where

*P*_*jnmi*, *k*_ = probability of person *j* receiving a rating *k* on item *i*;

*P*_*jnmi*, (*k*−1)_ = probability of person *j* receiving a rating *k−1* on item *i;*

*θ*_*j*_ = clarity measure of person *j*;

*δ*_*i*_ = difficulty of endorsing item *i*;

*τ*_*k*_ = difficulty of endorsing category *k* relative to *k−1, that is,* category threshold estimate;

*ν*_*nm*_ = clarity measures for demographic or grouping variables, including *research team*, *gender*, *ethnicity*, *racial group*, *academic rank title*, and *whether or not having interdisciplinary experience before*.

The ratio between *P*_*jnmi*, *k*_ and *P*_*jnmi*, (*k*−1)_ is called odds so that the log-odds (logits) is expressed as a linear combination of latent measures for different facets. In our analysis, there are eight facets including items, team members, research teams, gender, ethnicity, race, academic rank, and interdisciplinary experience. Since all the measures are on a common scale with logits as the units, the MFRM measures are on an interval scale, and the measures are additive. Higher logit values reflect higher clarity for individual members, research teams, or demographic groups. These facets are included with a positive sign in the model equation. The item facet and the category threshold have a negative sign in the equation, so that higher logit values indicate more difficult to endorse an item or a response category.

### Research Questions

We will address the following research questions (RQ).

RQ1: Is the scale reliable based on the clarity items, and do items fit each of the scales representing the constructs?

RQ2: Are the measures reliable for individual members, and do members fit the clarity scale?

RQ3: What are the results of clarity measures for the research teams, and how do they compare with each other?

RQ4: What are the results of clarity measures for demographic and academic-related subgroups, and how do they compare with each other?

RQ5: Are the 5-point Likert response categories used in an expected fashion?

RQ6: Does any item function differentially across demographic and academic-related subgroups, and can clarity measures be fairly used for different subgroups?

### Analysis Plan

Given the purpose of the study, we calibrated the items comprising goal clarity, role clarity, and process clarity, and constructed a latent scale of clarity measures for members, research teams, and subgroups that differ by individual characteristics. In particular, the reliability of separation indices for item facet and person facet was obtained. This index shows how distinct the measures are along the latent scale (i.e., clarity). The fit indices are used to examine how well each individual person or item fits the latent scale. The rating scale structure of the Likert scale is examined to see if the Likert response categories are used in an expected fashion. The DIF analysis is performed to investigate if any item presents threats to the fairness of clarity measures.

The major purpose of fitting an MFRM is to construct a latent scale with invariant measures. The clarity measures are used for making comparisons between individual members, research teams, and across different demographic groups. A chi-square test can be conducted to examine if different teams or groups have significantly different clarity measures. A significant result indicates that the teams or groups have unique clarity measures that are distinct from each other.

## Result

**Fig. 3.**
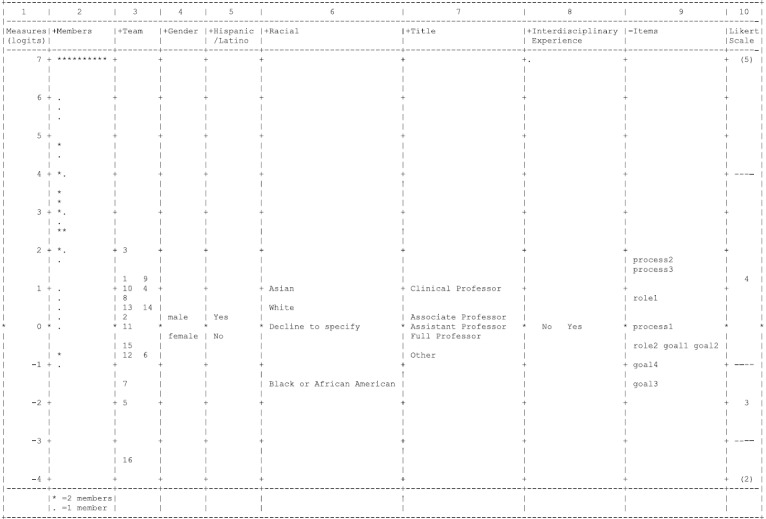
The Wright map.

The MFRM constructs a unidimensional scale for measuring clarity that maps all facets along a common continuum. The MFRM measures explain 67.60% of variances in item responses. Figure 3 shows the Wright map, which is an empirical display of the clarity scale. The first column displays the Rasch scores on logits scale. The items (column 9), members (column 2), research teams (column 3), and demographic and academic-related subgroups (columns 4–8) are located on the Wright map based on their Rasch scores. The last column (column 10) shows the threshold estimates of response categories on the Likert scale. Higher Rasch score of an item facet indicates it is more difficult for individual team members to endorse. A higher Rasch score of person-related facets indicates one has higher clarity measure. Since the measures have interval units (i.e., logits), we can easily compare the differences in clarity measures between individual members, research teams, and demographic groups. Next, we will address each research question based on the MFRM measures, Wright Map, category information, reliability, and fit indices.

### RQ1: Is the Scale Reliable Based on the Clarity Items, and Do Items Fit Each of the Scales Representing the Constructs?

Table 1 shows the observed mean values and Rasch scores for 9 items on the clarity scale. The observed average is the mean of raw rating responses to each item. The Rasch scores for items on the latent scale are centered at zero with a standard deviation of 1.08 logits. A higher Rasch score for an item corresponds to a lower observed mean score, indicating harder for respondents to agree with this item. The item process2 (i.e., *I know how my team will move forward with its work on our U-LINK project.)* has the highest Rasch score. We can see that the average score for item process2 is the lowest (4.29 out of 5) among all items, indicating fewer respondents agreed with this item compared with other items. Similarly, the item goal3 (i.e., *I know how my work relates to the overall objectives of my U-LINK team*) has the highest observed score (4.74 out of 5) and the lowest Rasch score (−1.48 logits). The item goal3 is the easiest one for respondents to agree with. Figure 3 displays the Rasch scores of 9 items (column 9). Based on the Rasch scores, it seems more difficult for respondents to attain higher clarity about team processes than team goals.

**Table 1.** Summary of Rasch scores for items

Item	Observed average (range: 1–5)	Rasch score		Infit MSE		Outfit MSE	
		Value	S.E.	Value	Fit category	Value	Fit category
goal1	4.64	−0.60	0.38	0.87	A	0.81	A
goal2	4.62	−0.46	0.38	1.43	A	1.50	A
goal3	4.74	−1.48	0.46	0.80	A	0.64	A
goal4	4.68	−0.92	0.41	1.16	A	0.90	A
role1	4.45	0.66	0.34	0.59	A	0.51	A
role2	4.62	−0.46	0.38	0.79	A	0.70	A
process1	4.55	0.07	0.35	0.92	A	1.18	A
process2	4.29	1.68	0.36	0.61	A	0.60	A
process3	4.43	1.50	0.44	1.88	C	1.87	C
*Mean*	4.56	0.00	0.39	1.00		0.97	
*SD*	0.14	1.08	0.04	0.42		0.46	

*Note*: (a) Observed average indicates mean score of all response ratings for each item. (b) Rasch score reflects the location of an item on the Rasch scale. Rasch scores are centered at zero with a standard deviation of 1.08 logits. (c) S.E. stands for standard error for each Rasch score. (d) The Infit and Outfit mean square error (MSE) are fit indices for identifying misfit items. (e) The fit category A (0.5 ≤ MSE ≤ 1.5) indicates an item is productive for measurement, and the fit category C (1.5 < *MSE* ≤ 2.0) shows an item is unproductive for measurement, but not distorting of measures. A full list of fit categories is provided in Appendix A2.

The reliability of separation for items is 0.87. The reliability index ranges from 0 to 1, and a value of 0.80 is generally considered as high reliability. It implies that the items can well represent the entire range of latent scale. The Infit and Outfit MSE for all the items, except Item process3, fall into a fit category of A, indicating a good fit of each item to the clarity scale. These items are productive for producing an invariant latent scale. Item process3 (i.e., *I am confident that my U-LINK team is using the right processes to move forward with its work.)* falls into the category C and is diagnosed as unproductive for measurement but not distorting. Both Infit and Outfit MSE values are relatively high for this item. A full list of fit categories is provided in Appendix A2.

### RQ2: Are the Measures for Individual Members Reliable, and Do Members Fit the Clarity Scale?

Individual members are ordered based on their clarity measures in the Wright map (Fig. 3, column 2). A higher Rasch score indicates one is clearer about the goals, roles, and processes of their research teams. Twenty out of 53 members have an observed average score of 5, meaning they have selected “agree” (5) on all items, excluding missing responses. The person measures are centered at 4.25 with a standard deviation of 2.70 logits. The reliability of separation for the member facet is 0.76. Without counting those individuals with extreme scores (i.e., all 5s), the reliability becomes 0.81, which is acceptable. The Infit and Outfit MSE can only be calculated for individuals who did not select 5 on all items. Among 33 individual members, three of them have an MSE above 2.0 (fit category D) based on Infit and Outfit statistics.

### RQ3: What Are the Results of Clarity Measures for the Research Teams, and How Do They Compare with Each Other?

Table 2 lists the Rasch scores for 16 research teams. They are also displayed on the Wright Map (Fig. 3, column 3). A higher measure indicates greater clarity. The team measures are centered at zero with a standard deviation of 1.41 logits. A chi-square test was conducted to examine if all teams share equal degree of clarity. Results showed that there were significant differences between 16 research teams, 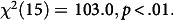 . Specifically, Team 3 reported the greatest clarity, and Team 16 scored the lowest on clarity. In addition, most teams fit the scale very well with a fit category of A. Only two teams (1 and 8) had relatively smaller Infit MSE values, indicating less productive for measurement but not distorting of measures.

**Table 2.** Team measurement report

Team	Rasch score		Infit MSE		Outfit MSE		# of valid responses	Response rate (%)
	Value	S.E.	Value	Category	Value	Category		
3	2.03	0.66	0.55	A	0.73	A	6	100.00
9	1.35	0.47	1.40	A	1.28	A	3	42.86
1	1.20	0.88	0.49	B	0.34	B	2	50.00
4	1.11	0.49	1.23	A	1.02	A	5	62.50
10	0.88	0.58	1.35	A	1.04	A	4	57.14
8	0.84	0.88	0.49	B	0.34	B	2	20.00
14	0.59	0.41	0.75	A	0.59	A	4	100.00
13	0.56	0.63	0.84	A	0.93	A	4	100.00
2	0.28	0.64	1.17	A	1.61	A	4	57.14
11	0.11	0.74	0.72	A	0.61	A	2	22.22
15	−0.61	0.38	0.60	A	0.51	A	4	100.00
12	−0.70	0.42	0.96	A	1.12	A	4	80.00
6	−0.80	0.34	1.10	A	0.98	A	5	55.56
7	−1.52	0.55	0.50	A	0.47	B	1	20.00
5	−1.94	0.51	1.18	A	1.54	A	1	16.67
16	−3.38	0.52	1.42	A	1.20	A	2	100.00
*Mean*	0.00	0.57	0.92		0.89			
*SD*	1.41	0.16	0.35		0.40			

*Note*: (a) Rasch scores are centered at zero with a standard deviation of 1.41 logits. (b) S.E. = Standard Error. (c) MSE = Mean Square Error. (c) The fit category A (0.5 ≤ MSE ≤ 1.5) indicates an item is productive for measurement, and the fit category B (MSE ≤ 0.5) shows an item is less productive for measurement, but not distorting of measures. A full list of fit categories is provided in Appendix A2. (d) The response rate of each team is provided in the last column. Please note that individual members who did not respond to any of the items or did not report complete demographic information were removed from the analysis. The total number of valid responses is 53 from 16 research teams.

### RQ4: What Are the Results of Clarity Measures for demographic and Academic-Related Subgroups, and How Do They Compare with Each Other?

The Wright Map displays the Rasch scores for each group (Fig. 3, columns 4–8). Chi-square tests were conducted to examine if the measures were different among different groups defined by demographic or grouping variables, including *gender*, *ethnicity, race*, *academic rank title*, and *whether or not having interdisciplinary experience before*. For all person-related facets, a higher measure indicates higher clarity. The measures of each grouping variable are centered at zero for the ease of group comparisons. Table 3 presents a summary of the chi-square test results.

**Table 3.** Summary of difference tests with Chi-square statistics

Grouping variables	Group sizes	Chi-square statistic	d.f. (*M* − 1)	p-value
Gender	Male: 26	3.30	1	0.07
	Female: 27			
Ethnicity group	Hispanic or Latino: 10	0.50	1	0.50
	Not Hispanic or Latino: 43			
Racial group	Asian: 6	16.00	3	<0.01
	Black or African American: 3			
	White: 42			
	Decline to specify: 2			
Academic rank	Assistant Professor: 13	16.90	4	<0.01
	Associate Professor: 15			
	Full Professor: 12			
	Clinical Professor/Professor of Practice/Research Professor: 7			
	Others: 6			
Interdisciplinary Experience	No: 19	0.50	1	0.47
	Yes: 34			

*Note*: (a) d.f. refers to degrees of freedom. (b) *M* refers to the number of groups defined by each variable.

The male group had higher clarity measure (0.24 logits) than the female group (−0.24 logits); however, this difference is not statistically significant. There is also a non-significant difference in clarity measures between Latino (0.13 logits) and non-Latino groups (−0.13 logits). In addition, the clarity measures of those who had interdisciplinary experience are similar to those who did not.

There is a significant difference in clarity measures across racial groups. The respondents included in our analyses are from three racial groups – Asian, White, and Black or African. The Asian group has the highest clarity measure (0.90 logits), followed by the Whites (0.47 logits). The Black or African American group has the lowest clarity measure (−1.40 logits), and it is lower than those who declined to specify their racial group. Besides, there is a significant difference between individuals with different academic ranks in their clarity measures. The Clinical Professor or Professor of Practice or Research Professor group has the highest clarity measure (0.98 logits). The associate professors rank the second with a measure (0.25 logits) higher than Assistant professors’ (−0.06 logits) and Full professors’ (−0.32 logits) clarity scores. Those who indicated other titles have the lowest measure (−0.86 logits).

### RQ5: Are the 5-Point Likert Response Categories Used in an Expected Fashion?

**Fig. 4.**
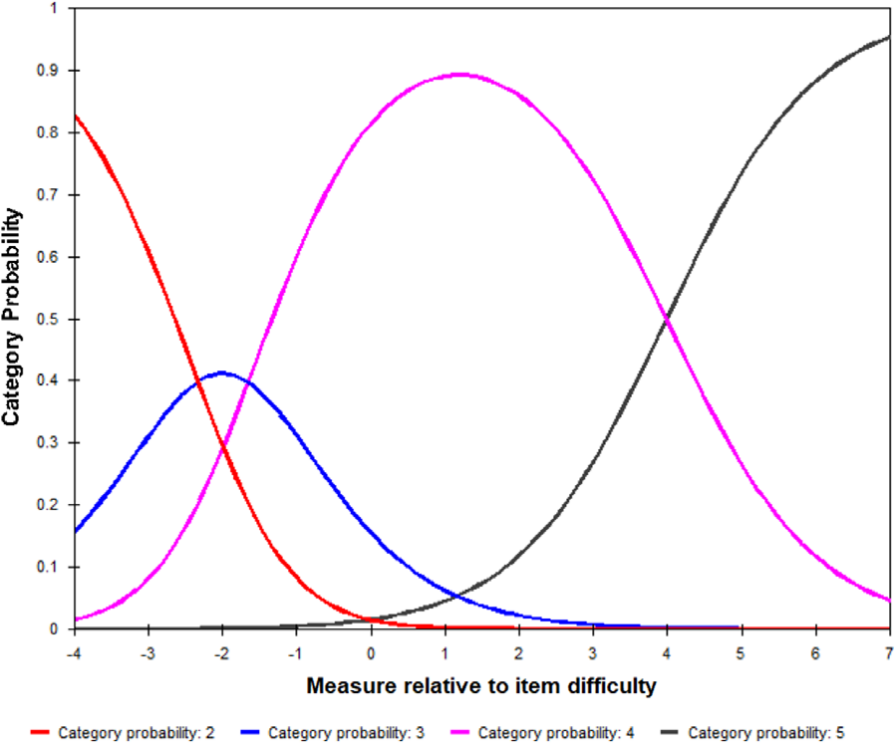
Category probability curves. *Note*: The vertical axis of probability function curves shows the probability of using a specific category, and the horizontal axis displays a continuum of person’s clarity measures relative to the item difficulty scores (*θ*_*j*_ − *δ*_*i*_). Different categories are color coded.

The Likert scale includes five categories ranging from 1 (Disagree) to 5 (Agree). An analysis of category responses (Table 4) indicates that no one used the score category of 1. The majority of responses were 4 (Somewhat agree; 42%) and 5 (Agree; 46%). In general, the usages of response categories conform to a cumulative scale such that an individual with a higher clarity measure is more likely to choose a higher score category (Fig. 4). There is no curve for Category 1 since no individual selected this category. The probability function curves of Category 4 and Category 5 are much wider than those of Category 2 and Category 3, confirming the much greater endorsement of the clarity items. Since all items are constrained to the same scale structure, based on the model specification, the probability function curves are in the same shape across all items. The threshold measures are increasing for higher score categories. The Outfit MSE reveals a good fit of category usages to the latent scale (between 0.5 and 1.5).

**Table 4.** Response category usages

Category	Proportion of usage (%)	Threshold measure		Outfit MSE	
		Value	S.E.	Value	Fit category
1. Disagree	0	–	–	–	–
2. Somewhat disagree	5	–	–	0.70	A
3. Neutral	7	−2.33	0.36	0.90	A
4. Somewhat agree	42	−1.66	0.28	1.00	A
5. Agree	46	3.98	0.17	1.00	A

*Notes*: (a) The proportion of usage in category 1 is 0% so that Rasch model does not provide any estimate for this category. (b) The threshold measures are the difficulty of endorsing category *k* relative to *k-1*. They are indicated as the locations (i.e., Rasch scores) where the adjacent category curves cross in Fig. 4. (c) The fit category A (0.5 ≤ MSE ≤ 1.5) indicates an item is productive for measurement.

### RQ6: Does Any Item Function Differentially Across Demographic and Academic-Related Subgroups, and Can Clarity Measures Be Fairly Used for Different Subgroups?

We evaluate the measurement invariance of latent measures across different demographic groups, including *gender*, *ethnicity*, *race*, *academic rank title*, and *whether or not having interdisciplinary experience before*. DIF analyses are preformed separately on each demographic variable. The *t*-tests are used for comparing the differences in the Rasch scores of items between different groups. If a significant difference is found for an item, this item shows DIF and presents potential threats to the fairness of the clarity scale.

Table 5 shows the analysis results for the identified DIF items between certain demographic and academic-related subgroups. Among nine items on the clarity scale, two of them exhibit significant DIF. Specifically, DIF occurs on Item process3 (*I am confident that my U-LINK team is using the right processes to move forward with its work.*) between gender groups. For members with the same clarity measures, this item is more difficult for males to endorse, compared to females. The DIF effect is large based on the contrast value (i.e., 2.55). Item goal2 (*I am confident that I know what the goals are for my U-LINK team.)* shows DIF between those with and without interdisciplinary experiences. Therefore, among members who are at the same clarity level, it is more difficult for those who had no interdisciplinary team experience before to agree with this statement than those with experiences. Item Goal 2 also has a large DIF effect given the contrast value (i.e., −2.03). DIF is not detected for any item between ethnicity and racial groups, as well as subgroups with different academic rank titles.

**Table 5.** Differential item functioning (DIF) analysis results between demographic and other subgroups

**Panel A: DIF between gender groups**								
Item	Group 1: Male		Group 2: Female		*t*-test for difference			Effect size
	Measure	S.E.	Measure	S.E.	*t*	d.f.	p-value	DIF contrast
process3	3.00	0.74	0.45	0.60	2.67	18	0.02	2.55
**Panel B: DIF between groups with and without interdisciplinary experience**								
Item	Group 1: With interdisciplinary experience		Group 2: Without interdisciplinary experience		*t*-test for difference			Effect size
	Measure	S.E.	Measure	S.E.	*t*	d.f.	p-value	DIF contrast
goal2	−1.43	0.60	0.40	0.50	−2.32	30	0.03	−2.03

*Note*: (a) d.f. refers to degrees of freedom. (b) The DIF contrast (i.e., the difference of item measures between two groups) serves as the effect size. An item shows slight to moderate DIF when an absolute value of DIF contrast is greater than 0.43, and it presents moderate to large DIF when the contrast is greater than 0.68 [31].

## Discussion and Limitation

Although there is a need to identify common set of measures for team sciences, it is even more crucial to ensure the measures are valid, reliable, and fair. Our study outlines the procedures for constructing and examining team science measures and further illustrates this process through an empirical example based on the clarity measures of interdisciplinary scientific teams’ collaboration processes. This approach to scale development and assessment allows revisions to established scales and yields objective measures that can be used across interdisciplinary teams with diverse compositions, or which are located at different institutions.

**Fig. 5.**
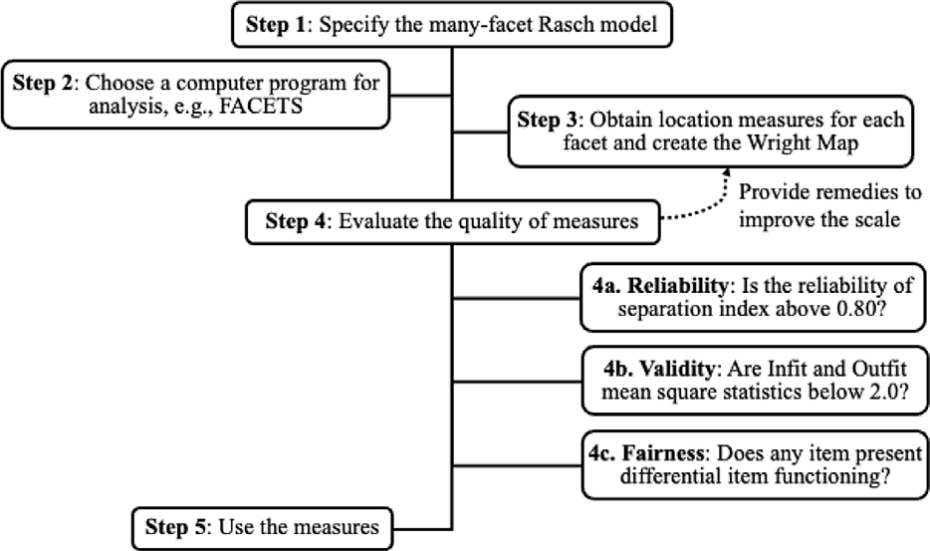
Scale development procedure using many-facet Rasch model.

In this study, we demonstrated how a many-facet Rasch model can be used to examine and evaluate team science measures. The process of developing a scale using many-facet Rasch model is presented as a flow chart in Fig. 5. We first specified our model given the observed data responses and the facets of interest (e.g., individuals, research teams, items). Then, we chose a computer program for running the analysis, for example, the FACETS computer program that is specialized for fitting Rasch models with more than two facets. The output results provided us Rasch scores of each facet that can be used to create an empirical scale – the Wright Map, as well as various indices for evaluating the quality of the measures. Once the quality of all measures was satisfactory, they were used for making inferences and decisions. In our empirical analysis, we provided comparison between research teams as well as demographic and academic-related subgroups. These measures can also be used for creating norm scales, setting standards, or generating profiles for members and teams.

Based on our evaluation of the clarity scale in the empirical example, we found an item that is not fit well to the scale – process3. This item had concerning high Infit and Outfit indices as well as showed DIF between gender groups. An examination of missing responses revealed that 33% of members did not respond to this item. This missing rate was very high, especially compared to other items. There was just one missing response to Item process2, and there were no missing on all other items. Content specialists should be consulted in terms of future uses of Item process3 for measuring clarity.

In addition to item process3, item goal2 had DIF between teams with and without interdisciplinary experience. In particular, item process3 may cause bias in scores against the male respondents, and item goal2 may be biased against those without prior interdisciplinary experience. DIF can only detect statistical bias. To confirm the unfairness of an item, we need to investigate why the groups differ in their probabilities of agreeing with this item [22]. For example, it may be because of differing understanding of a word or phrase [23]. Further screening of item content, interviews with respondents, or analysis of process data can help us unravel the reasons. In the meantime, the DIF items should be revised or removed for future uses. The investigation of DIF items may also help clarify the definition of the measuring construct and eventually strengthen the quality of instrument.

Three members (of 33 team members) had unexpected responses that may affect the validity of the Rasch scores and may have produced distorted measures. Further analyses involving qualitative approaches can be used to investigate reasons that why these three members established unexpected response patterns. Additionally, Team 1 and Team 8 had low fit indices, and this may be due to a lack of variation in their responses.

Based on the usage of rating categories, the item statements seem to be relatively easy for respondents to endorse. In other words, team members tend to agree with most of the items. Figure 3 (column 10) shows that the majority of members have higher Rasch scores (above 2 logits) than almost all of the items (below 2 logits). With a positive difference in logits (i.e., person measure – item measure), the individual member has a higher probability of endorsing an item or choosing a higher score category. This means that the current instrument for measuring clarity may not be sufficient in separating teams with high clarity levels. To improve the quality of instrument, future researchers should consider including items that reflect higher levels of clarity in each aspect or adding more categories at the higher end to distinguish different degrees of agreement toward the statements.

We encountered several challenges in our data analyses. At first, our analyses showed that about 37.74% of respondents selected “agree” (5) on all items, generating extreme scores. The large proportion of extreme scores constitutes a lack of variation in responses, and this poses difficulty for measurement models to be estimable and to produce valid measures. In our empirical illustration, reliability of our measure was reduced when large number of extreme scores were included. This is usually called as “ceiling effect” in self-report survey data.

Researchers and practitioners who construct assessments for measuring team science-based constructs should consider including items that reflect greater difference in the levels of the latent variable. For instance, more “extreme” items that reflect a higher level of clarity can be used to increase the range of the scale. In addition, using more categories of response options may be helpful for reducing ceiling effects [24–26]; however, Keeley *et al.* [27] indicated that adding categories did not help.

**Fig. 6.**
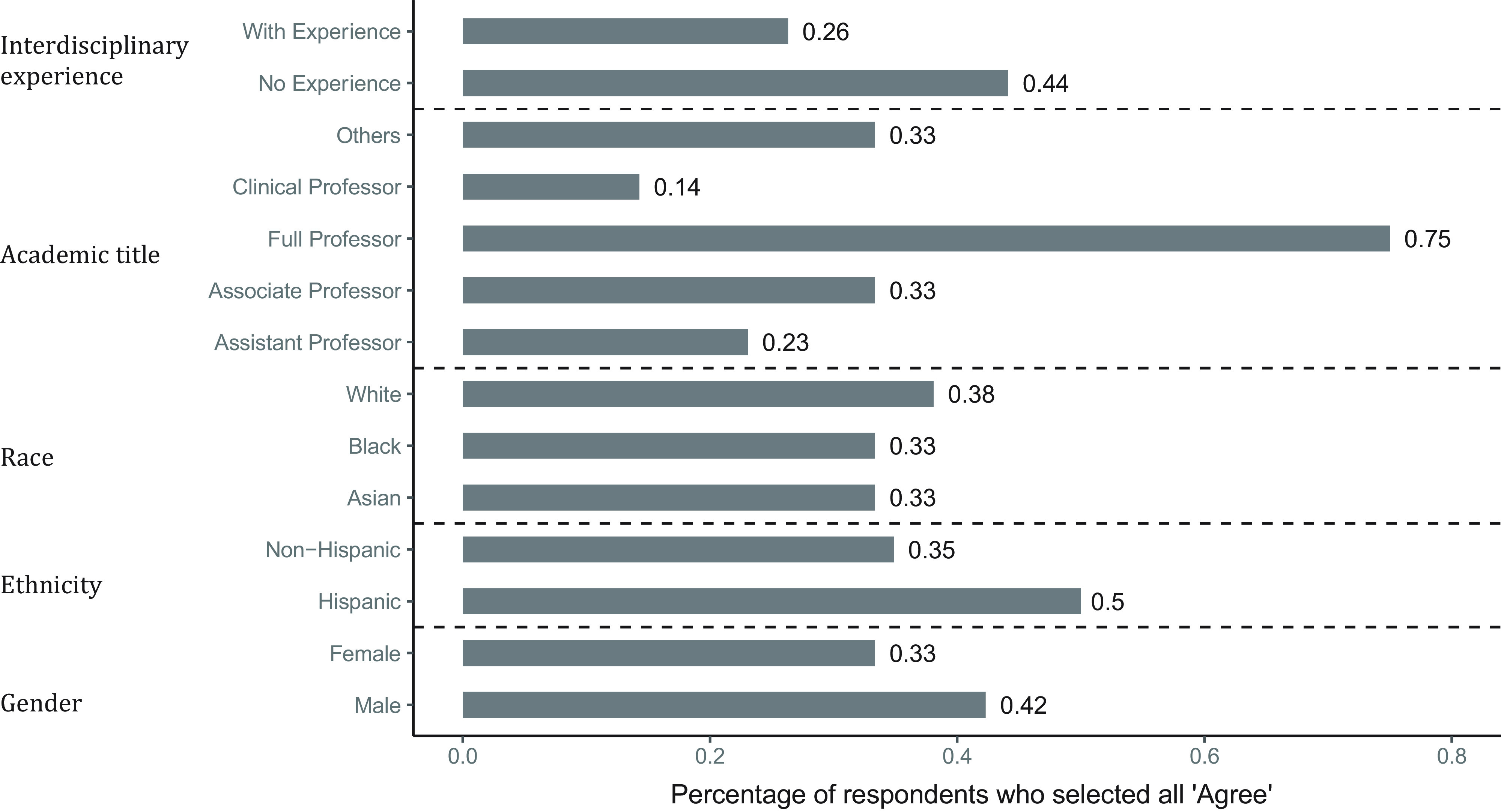
Characteristics of respondents who selected all “agree.” *Note*: The number besides the bar shows the actual percentage of extreme scores (rating of 5) in each group.

Ceiling effects have been found to vary across different age groups [28], which implies that they may be associated with personal characteristics. We examined the characteristics of the team members. Figure 6 shows the percentage of members who selected “agree” (rating of 5) on all items in each demographic or academic group. We discovered that among 20 respondents with extreme scores, 25% were non-Hispanic and White full professors with prior interdisciplinary experience. We also found that 67% of team members in Team 3 had extreme scores, which is much higher than other teams. This implies that the ceiling effects may also be associated with team characteristics.

Second, due to the removal of missing responses either on the clarity items or demographic questions, the number of responses available reduced dramatically in some research teams. For instance, Team 5 has six members, but only one person responded to the clarity questions. Team 8 has 10 members, but the responses of only two team members were complete. Limited data from members of a group cannot fully represent the team functioning, and uneven team sizes may also influence the measurement results. To improve the measurement results, a larger sample size with more variant responses can enhance the quality of the latent scale, and more actions should be taken to reduce missing responses.

Third, the U-LINK project provided funding for research teams in phases, with the allocation of subsequent funding being linked to team success (e.g., productivity). However, the survey was distributed before funding decisions were made so the assessment survey was not a factor for making funding decisions. Nonetheless, individual members may have provided more positive responses in order to present a better image of their teams. This is a common issue in self-reported measures, and it poses another challenge for administering team science assessments and obtaining accurate responses. Measurement models can be helpful to identify outlying individuals who provided unexpected responses; however, more proactive approaches should be considered to minimize any potential response bias. An alternative approach is to use the third-party raters to conduct observations of team functioning and provide their ratings. Our proposed quantitative approach based on the many-facet Rasch model can also be used to evaluate rater-mediated assessments.

## Future Directions

Future studies should explore the use of a multidimensional Rasch model for examining team science measures. The current study employed an empirical analysis to construct a unidimensional scale with a single latent continuum. When multiple constructs are present, multidimensional Rasch models can be used following the proposed scale development procedures. It is worth noting that a multidimensional scale requires a sufficient sample size and enough number of items for measuring each dimension. For instance, the required sample size is usually bigger than those required for the creation of a unidimensional scale. However, one contribution of modern measurement theories including Rasch measurement theory is the ability to create a norm scale that can be used to compare individuals who complete psychological assessments at different time points. Future studies should focus on the creation of norm scales for team science measures. Future studies should also examine the association of ceiling effects, especially those that are related to team characteristics. Given that significant differences of clarity measures were found between racial groups and faculty in different academic ranks, future studies can also explore the reasons behind the significant differences by using qualitative approaches such as interviews with team members.

To improve the psychometric quality of latent scores in this study, we focused on the validity, reliability, and fairness evidence that are stressed by the *Standards for educational and psychological testing* [14] as foundational areas. In future studies, a fourth component – comparability [29] – should be considered as part of the process of evaluating the soundness of team science measures. Comparability relates to the linking of different scales for producing comparable scores so that team members measured by different instruments with subsets of items can be compared against each other or against a common standard [30]. In the team science field, not all items are likely to be appropriate for assessing team functioning across different disciplines, time, institutions, or geographic regions. However, we often want to compare the performance of these research teams across institutions and team compositions. In this case, instruments can be constructed with different versions of questionnaires to better suit the needs of various teams. As long as a few good-quality common items are included in all forms, techniques based on Rasch measurement theory can be used to link different scales to ensure comparability across person and team scores. Future studies should investigate and apply appropriate quantitative methods for linking scales to create comparable measures of team science processes and outcomes. Furthermore, based on Rasch measurement models, an item bank can be created and maintained for implementing computerized adaptive testing.

## Conclusion

The current study is the first attempt to demonstrate the use of Rasch measurement theory to examine the psychometric properties of team science measures. Of many strengths, many-facet Rasch models can be used to (a) produce invariant measures on a latent continuum, (b) assess the validity, reliability, and fairness of latent measures, and (c) facilitate objective comparisons between individual members, research teams, and various demographic and academic-related groups. The characteristics of Rasch scores offer a number of practical applications, which will advance our understanding of the dynamics of interdisciplinary scientific teams.

First, standardized scores can be obtained using Rasch models for individual team members. For instance, the empirical example in this study shows how we obtained objective measures of clarity about goals, processes, and roles in an interdisciplinary team. With standardized scores of team science measures, we can create individual profile showing interdisciplinary team experiences including contribution and readiness. This information can be further utilized for various purposes at the institutions, such as promotion and tenure, merit-based awards and incentives, and scholarly visibility.

Second, at the team level, standardized scores can provide objective comparisons across research teams and further serve as quantitative feedback for teams to identify areas that can be targeted for improvement by team development and training efforts, including workshops or other trainings that support the success of interdisciplinary teams. We can create norm scales using these standardized scores to assess how a team changes over time and how it compares with others. Regular “check-ups” using these questionnaires can provide real-time information to individual members regarding their status in interdisciplinary scientific teams and diagnostic feedback for teams and team members to work together to improve their performance in handling conflicts and improving clarity.

Third, given that many institutions have initiated internal funding programs for promoting interdisciplinary research, Rasch scores can be used to evaluate program success regularly and further track outcomes over time at the institution.

Finally, Rasch measurement theory can enable SciTS researchers to develop and utilize invariant and high-quality measures across different disciplines, institutions, and cultures and thus empirically test the underlying model using advanced statistical analysis. Because we anticipate that research efforts focused on SciTS will continue to grow, establishing a process of creating standardized measures will also help synthesize empirical study findings in the future.

## Supplementary material

For supplementary material accompanying this paper visit https://doi.org/10.1017/cts.2022.472. click here to view supplementary material
