# Supplementary material for: Measuring the processes of interdisciplinary team collaboration: Creating valid measures using a many-facet Rasch model approach
Source: J Clin Transl Sci. 2022 Oct 6;6(1):e134. doi: 10.1017/cts.2022.472 (PMC9794954; doi:10.1017/cts.2022.472)
Supplement: Supplementary file 1 [file S2059866122004721sup001.docx]

Appendix A1 – Item statements and demographic variables

| **Items** | **Statement** |
| --- | --- |
| goal1 | I am clear about my responsibilities on this U-LINK team. |
| goal2 | I am confident that I know what the goals are for my U-LINK team. |
| goal3 | I know how my work relates to the overall objectives of my U-LINK team. |
| goal4 | I know how my work will contribute to the ultimate goals of my U-LINK team. |
| role1 | I know exactly what is expected of me on my U-LINK team. |
| role2 | I know what my responsibilities are on my U-LINK team. |
| process1 | I know how to go about my work on my U-LINK team. |
| process2 | I know how my team will move forward with its work on our U-LINK project. |
| process3 | I am confident that my U-LINK team is using the right processes to move forward with its work. |
| **Variables** | **Values** |
| Team number | 1-16 |
| Gender | 1 = male  2 = female |
| Ethnicity group | 1 = Hispanic or Latino  2 = Not Hispanic or Latino |
| Racial group | 1 = Asian  2 = Black or African American  3 = White  4 = Decline to specify |
| Academic rank | 1 = Assistant Professor  2 = Associate Professor  3 = Full Professor  4 = Clinical Professor/Professor of Practice/Research Professor  5 = Other |
| Interdisciplinary Experience | 0 = No  1 = Yes |

Appendix A2. Fit categories for interpreting infit and outfit mean square errors

| Mean Square Residual (MSE) | Interpretation | Fit Category |
| --- | --- | --- |
| $0.5\leq\mathrm{MSE}\leq1.5$ | Productive for measurement | A |
| $\mathrm{MSE}<0.5$ | Less productive for measurement, but not distorting of measures | B |
| $1.5<\mathrm{MSE}\leq2.0$ | Unproductive for measurement, but not distorting of measures | C |
| $2.0< \mathrm{MSE}$ | Unproductive for measurement, distorting of measures | D |

Appendix B – FACETS syntax for empirical data analysis

Title = SciTS (U-LINK data) Goal, process, and role clarity

arrange = m

facets = 8

positive = 1, 2, 3, 4, 5, 6, 7

dvalues = 8, 1-9

gstat=yes

model=?,?,?,?,?,?,?,?,R5

*

Labels=

1, Members

1-97 =

*

2, Team

1-16 =

*

3, Sex

1 = male

2 = female

*

4, Latino

1 = Yes

2 = No

*

5, Race

1 = American Indian or Alaska Native

2 = Asian

3 = Black or African American

4 = Native Hawaiian or Other Pacific Islander

5 = White

7 = Decline to specify

*

6, Title

1 = Assistant Professor

2 = Associate Professor

3 = Full Professor

4 = Instructor

5 = Clinical Professor/Professor of Practice/Research Professor

6 = Other

*

7, Interdisciplinary Experience

1 = Yes

2 = No

*

8, Items

1 = goal1

2 = goal2

3 = goal3

4 = goal4

5 = role1

6 = role2

7 = process1

8 = process2

9 = process3

*

Data =
